# Design, Electron Transfer Process, and Opto-Electronic Property of Solar Cell Using Triphenylamine-Based D-π-A Architectures

**DOI:** 10.3390/ma12010193

**Published:** 2019-01-08

**Authors:** Yuanchao Li, Lu Mi, Haibin Wang, Yuanzuo Li, Jianping Liang

**Affiliations:** 1 College of Science, Northeast Forestry University, Harbin 150040, China; liyuanchao2018@126.com (Y.L.); milufine@sina.com (L.M.); whbhit@126.com (H.W.); 2 Key Lab of Materials Modification, Ministry of Education, Dalian University of Technology, Dalian 116024, China

**Keywords:** opto-electronic property, dye-sensitized solar cells, density functional theory, electronic structure

## Abstract

A series of D-π-A type dyes were designed based on the experimentally synthesized A1 by introducing different functional groups on the donor and π-spacer, and the optical and electrical properties were calculated by using density functional theory (DFT) and time-dependent DFT (TD-DFT). P1–P6 present highest light harvesting efficiency (LHE), driving force of electron injection ($\Delta G^{inject}$), reorganization energy ($\Delta G_{reg}$) and $eV_{OC}$. These critical parameters have a close relationship with the short-circuit current density ($J_{SC}$) and open-circuit photovoltage ($V_{OC}$), and lead to P1–P6 will exhibit higher efficiency. D4 also exhibit superior properties in the driving force of electron injection ($\Delta G^{inject}$), reorganization energy ($\Delta G_{reg}$), which will lead to a higher short-circuit current density ($J_{SC}$). We hope that these results will be helpful for experiments to synthesize new and highly efficient dyes.

## 1. Introduction

Efficient development and utilization of renewable energy is an important way to solve energy shortage. Solar energy has received wide attention compared with other renewable energy sources due to its unlimited supply. Since Grätzel and O’Regan reported dye-sensitized solar cells (DSSC) in 1991 [1], it is quickly regarded as an alternative to silicon solar cells because of their easy production processes and lower production prices [2,3,4,5,6,7]. Dye sensitizer is a key part of DSSC to improve performance, and a series of dyes have been studied including porphyrin dyes, Ru-based dyes (such as N3/N719 [8] and the black dyes [9]) and metal-free organic dyes. Ru-based dyes and porphyrin dyes are regarded as promising sensitizers, and the power conversion efficiency (PCE) have reached 13% [10]. However, it is not ideal for large-scale production due to high cost, not being environmentally friendly, and a complex synthesis and purification process [11,12]. Therefore, metal-free organic dyes have become an alternative to Ru-based dyes and porphyrin dyes for their high molar extinction coefficient, low cost, and ease of synthesis [13,14,15,16,17].

The literature reports many different structures organic sensitizers including D-A, D-π-A, D-π-A-π-D, D-D-π-A, D-A-π-A, and D-π-π-A [18,19,20,21,22,23,24]. Among them, the most metal-free organic dyes have donor-π-acceptor (D-π-A) structure [25], mainly including prophyrin dyes [26,27], coumarin dyes [28,29], triphenylamine dyes [30,31], indoline dyes [32,33], and so on. The D-π-A structure with acceptor (such as sulfonic acid, cyanoacrylic acid, phosphonic acid, and carboxylic acid) binds to the semiconductor (TiO_2_) surface, which will facilitate electron transfer to the TiO_2_ and the regeneration of excited dyes to the ground state by the redox shuttle [14]. Metal-free organic dyes with D-π-A structure have been widely used in DSSC due to the above advantages [34,35]. Among reported D-π-A structure, triphenylamine and cyanoacrylic acid were regarded as excellent donor and acceptor units [36,37]. Previous research indicates that dye with D-π-A structure can efficiently promote intramolecular charge transfer from the donor to acceptor moiety, which will lead to broadening the absorption spectrum and increasing the charge carrier mobility [38,39]. In order to achieve high power conversion efficiency, the dyes should meet the following requirements [40], dyes should have enough driving force to effective electron injection, and the oxidation potential must be lower than that of redox potential of $I^{-}/I_{3}^{-}$ electrolyte, which facilitates oxidation dye regeneration. There should be a strong electronic coupling between the dye acceptor and the semiconductor substrate, which ensures charge transfer channel perfect electron injection. The absorption spectrum should have a strong absorption in the region of 400–600 nm. Recently, Chen et al. [41] synthesized two novel dyes CC201 and CC202, they exhibit strong response in the region of 500−800 nm. CC202 exhibits higher power conversion efficiency (PCE) of 6.1% with cobalt electrolyte. Kim et al. [42] designed two D-π-A dyes IAH and IDH to study the effect of the alkyl chain location at different positions on the photoelectric properties. IAH exhibits more electron injection from the excited dye to the conduction band of TiO_2_, which leads to a high short-circuit photocurrent ($J_{SC}$). IDH more effectively inhibits electron transfer from the TiO_2_ conduction band to the excited dye, leading to a high open-circuit photovoltage ($V_{OC}$). IAH showed a higher power conversion efficiency (PCE) of 6.90% compared to IDH. Li et al. [24] designed a series of dyes with different π-spacer and acceptor groups. The results showed that the longer conjugated bridge would inhibit the intramolecular charge transfer (ICT). The dye with (E)-3-(4-(benzo[c][1,2,5] thiadiazol-4-yl) phenyl) -2-cyanoacrylic acid (TCA) acceptor group has the higher photoelectrical property due to the lowest chemical hardness, largest electroaccepting power, dipole moment, and the change in the energy of the TiO_2_ conduction band among the designed dyes. Panneerselvam et al. [43] designed seven TPA molecules with different π-linkers based on two experimentally reported molecules. The results show that furan moieties bring planarity to the molecule and exhibit lower HOMO-LUMO gap. TPA9 exhibits highest absorption maxima. TPA7 has high emission maxima and exhibit planarity in excited state. There is an obvious correlation between optoelectronic properties and HOMO-LUMO gaps. Biswas et al. [44] designed a series of dyes with different π-spacer units. The results show that dye 3 with fused-pyrrole as a spacer will exhibits high efficiency compared to dye 1. The better short-circuit photocurrent ($J_{SC}$) of dye 3 would be higher than dye 1 due to higher driving force of electrons, singlet excited state lifetime, and the maximum absorption wavelength. Dye 3 will have better open-circuit photovoltage ($V_{OC}$) than dye 1 due to larger dipole moment.

**Figure 1:**
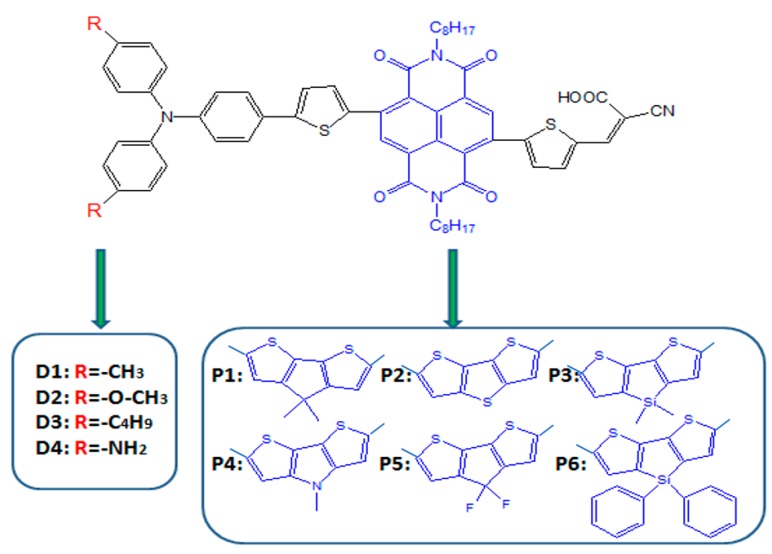
Chemical structure of dyes.

To reach high efficiency, R. Bobe et al. [45] introduced simple triphenylamine as electron donor, naphthalenediimide (NDI) as the π-spacer embedded between the two thiophene units and cyanoacrylic acid as the electron acceptor to design and synthesize a new sensitizer A1 based on R1, and the molecular structure is shown in Figure 1. The power conversion efficiency (PCE) of A1-sensitized DSSC device reached 6.24, 6.05, and 5.17% using cobalt-, solvent-, and ionic liquid-based redox shuttles, respectively. At the same time, chemical modification on individual units (such as donor, π-spacer and acceptor) is one of the important ways to improve efficiency of DSSCs through the theoretical calculation. Hence, we designed a series of dyes (Figure 1) based on A1 by modifying the donor, π-spacer and acceptor groups. In this work, our aim is to study the influence of tune the A1 structure on the photoelectric parameters, and further, to find good candidates for DSSC applications.

## 2. Computational Details

### 2.1. Theoretical Background

As we have known that the sunlight-to-electricity conversion efficiency (η) of DSSCs device is determined by the open-circuit photovoltage ($V_{OC}$), the short-circuit photocurrent ($J_{SC}$), the fill factor (FF) and incident solar power on the cell ($P_{inc}$). η can be described by [46]

(1) $$\eta =FF\frac{V_{OC}J_{SC}}{P_{inc}}$$

In DSSCs, $J_{SC}$ can be determined by the following equation [47]

(2) $$J_{SC}=\int_{\lambda }LHE(\lambda )\phi_{inject}\eta_{collect}d\lambda$$

where LHE(λ) is the light harvesting efficiency at a given wavelength, $\phi_{inject}$ is the electron injection efficiency, and $\eta_{collect}$ denotes the charge collection efficiency. All the components of DSSCs are only different in dyes, hence $\eta_{collect}$ can be assumed to be a constant. LHE(λ) can be expressed as

(3) $$LHE=1-10^{-f}$$

where f represents the oscillator strength of dye molecules corresponding to wavelength $\lambda_{\max}$. $\phi_{inject}$ is related to injection driving force $\Delta G^{inject}$ of electrons injecting from the excited dyes to the semiconductor substrate. $\Delta G^{inject}$ can be estimated as [48,49]

(4) $$\Delta G^{inject}=E^{dye*}-E_{CB}$$

where $E^{dye*}$ is the oxidation potential of the excited dye and $E_{CB}$ is the conduction band edge of the semiconductor ($E_{CB}$ = −4.00 eV). $E^{dye*}$ can be estimated as [50]

(5) $$E^{dye*}=E^{dye}-E_{0-0}$$

where $E^{dye}$ is the redox potential of the ground state of the dye and $E_{0-0}$ is the vertical transition energy associated to the $\lambda_{\max}$. Hence the larger LHE and $\Delta G^{inject}$ are benefit to increase $J_{SC}$ value.

In DSSCs, $V_{OC}$ can be expressed by the following equation [50]

(6) $$V_{OC}=\frac{E_{CB}+\Delta E_{CB}}{q}+\frac{k_{b}T}{q}\ln(\frac{n_{c}}{N_{CB}})-\frac{E_{redox}}{q}$$

where *q* is the unit charge, $E_{CB}$ is the conduction band edge of the semiconductor substrate, $k_{b}T$ is the thermal energy, $n_{c}$ is the number of electron in the conduction band, $N_{CB}$ is the density of accessible states in the conduction band and $E_{redox}$ is the electrolyte Fermi level. $\Delta E_{CB}$ is the shift of $E_{CB}$ when the dyes are adsorbed on substrate and it can be described by [51]

(7) $$\Delta E_{CB}=-\frac{q\mu_{normal}\gamma }{\varepsilon_{0}\varepsilon }$$

In this expression, $\mu_{normal}$ is the dipole moment of individual dye perpendicular to the surface of semiconductor substrate, γ denotes the surface concentration of dyes. $\varepsilon_{0}$ and $\varepsilon_{0}$ represent the vacuum permittivity and the dielectric permittivity, respectively. It is obvious that $\mu_{normal}$ is the key factor determining $V_{OC}$.

### 2.2. Computational Detail

**Figure 2:**
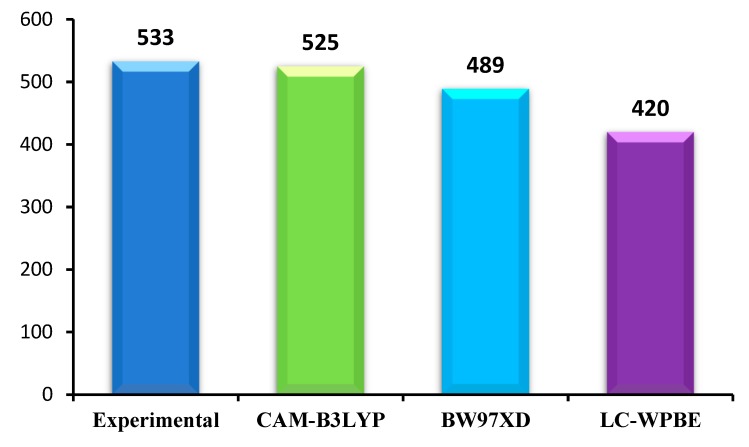
Maximum absorption wavelength ($\lambda_{\max}$/nm) of A1 in chloroform solution calculated by TD-DFT at different functionals with 6-31G(d) level.

The ground state geometries of all the dyes before and after binding to TiO_2_ in chloroform solvent were fully optimized using density functional theory (DFT) [52] at B3LYP [53] level, 6-31G(d) for nonmetal atoms, while LANL2DZ basis set for Ti atom. The excitation energies, oscillator strengths, and the UV–vis absorption spectra of all the dyes before and after binding to TiO_2_ in chloroform solvent were simulated using the time-dependent density functional theory (TDDFT) [54] with CAM-B3LYP [55] function, 6-31G(d) basis set for C, H, O, N, S atoms and LANL2DZ basis set for Ti atom on the basis of optimized geometries. However, different exchange–correlation (XC) functionals have a significant effect on the absorption spectra. In order to find a suitable functional, three different XC functionals (CAM-B3LYP, B3PW91, and LC-WPBE) combined with 6-31G(d) basis set are used to calculate absorption spectra in chloroform solvent. The conductor-like polarized continuum model (C-PCM) method is used to simulate the solvent effects. This method was recognized as access reliable results to the experimental values [56]. The calculated results are shown in Figure 2. From the Figure 2, it is obvious that CAM-B3LYP functional is close to the experimental values among five different XC functionals. Kathiravan et al. used CAM-B3LYP to predict the absorption spectra closer to the experimental values compared to other functional [57]. Many previous literatures have shown that CAM-B3LYP can accurately characterize UV–vis absorption spectra [44,58,59]. Therefore, CAM-B3LYP can provide a more reliable and rational description of the UV–vis absorption spectra, and it is used to simulate the absorption properties of dyes before and after binding to TiO_2_ in chloroform solvent. All calculations were performed with Gaussian 09 package [60].

## 3. Results and Discussion

### 3.1. Screening of the π-Spacer

The π-linker in D-π-A structured dyes is a key factor to affect charge transfer, light absorption and photophysical properties of dyes. R. Bobe et al. [45] find that the extension of π-linker via the insertion of NDI moiety between the two thiophene units exhibits superior performance. Therefore, we designed a series of D-π-A dyes, which triphenylamine as electron donor, cyanoacrylic acid as electron acceptor, and different thiophene derivatives were inserted between the two thiophene units as π-linker (see Figure 1). Commonly, the conjugation degree is an important parameter that affects the absorption property of the dyes. In order to study the influence of different π-spacers on the degree of conjugation, the ground state structures of dyes were optimized at DFT/B3LYP/6-31G(d) in chloroform solvent, and the selected parameters (bond length and bond angle) are listed in Table 1. The dihedral angles ($\Phi_{1}$) between the donor and π-spacer are −21.17, 21.30, −20.56, −20.53, −21.06, −18.34, and 22.46° for P1–P6 and A1, respectively. This clearly indicates that the dihedral angles ($\Phi_{1}$) of designed dyes P1–P6 are slightly smaller than that of A1, which means that the conjugation degree of P1–P6 are superior to A1. Hence, introduction of different thiophene derivatives to replace NDI moiety is not only enhance conjugation degree but also inhibit π-aggregation due to the slightly lower the dihedral angle ($\Phi_{1}$). The favorable conjugation of P1–P6 will increase the delocalization of frontier molecular orbitals (FMOs) and facilitates electron transfer process throughout the molecule. The dihedral angles ($\Phi_{2}$/$\Phi_{3}$) between the thiophene derivative and the thiophene are 9.48/0.20, −9.82/−1.96, 7.54/1.84, 8.76/0.35, 11.17/4.81, 8.75/1.33, and −37.79°/−118.77° for P1–P6 and A1, respectively. It is found that the dihedral angles ($\Phi_{2}$/$\Phi_{3}$) of P1–P6 are smaller than that of A1, indicating that after introduction of different thiophene derivatives to replace NDI moiety, the π-spacer more rigid. Generally, dyes with rigid π-spacer exhibit outstanding performance compared with corresponding twistable π-spacer. The dihedral angles ($\Phi_{4}$) between the π-spacer and acceptor are −179.94, −0.25, −179.72, 179.86, 179.93, −179.92, and −179.70° for P1–P6 and A1, respectively, which are all close to 0°. This means that donor and acceptor is more in a plan, thereby the excited electrons are more easily transferred from donor to acceptor and finally inject into the TiO_2_. Thus, introduction of different thiophene derivatives in π-spacer facilitates electron transition. Table 1 clearly shows that the bond lengths $d_{1}$ and $d_{4}$ of all dyes have no significant change with different π-spacer. The bond lengths $d_{2}$ and $d_{3}$ of P1–P6 are in the range of 1.441–1.442 and 1.433–1.438 Å, respectively. There is no obvious change. Compared to A1, the bond lengths $d_{2}$ and $d_{3}$ are relatively shortened with introduction of thiophene derivatives in the π-spacer (P1–P6). The shorter bond length is beneficial to facilitate the ICT in the D-π-A molecules [44]. The bond lengths of all dyes are in between C–C single and C=C double bonds.

**Figure 3:**
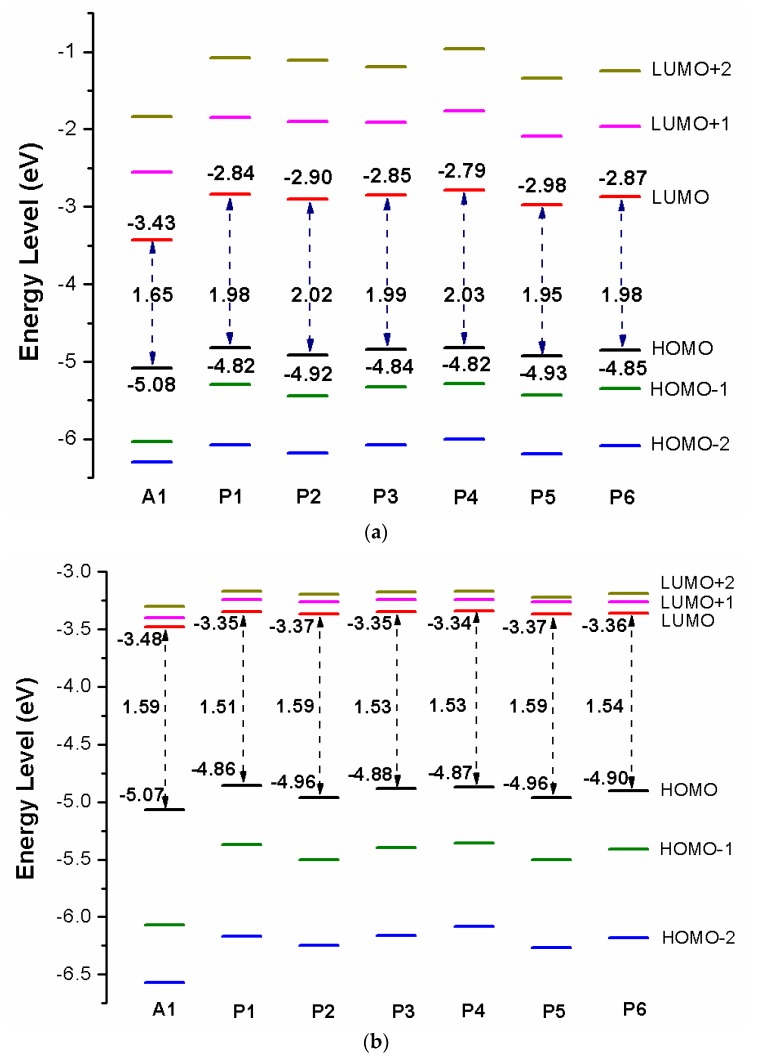
Energy level diagram of (**a**) isolated dyes P1–P6 and A1, (**b**) dyes P1–P6 and A1 after bound to TiO_2_ surface in chloroform solvent.

Over the years, the energy levels and frontier molecular orbitals (FMOs) are used to obtain information on the electronic excitation and transition properties of dyes [61,62]. The molecular orbital energy diagrams of HOMO−2, HOMO−1, HOMO, LUMO, LUMO+1, and LUMO+2 are computed at DFT/B3LYP/6-31G(d) in chloroform solvent and depicted in Figure 3a. To design effective dyes, the HOMO and LUMO levels of dyes must match to the redox potential of $I^{-}/I_{3}^{-}$ electrolyte and the conduction band (CB) of the TiO_2_ [36]. The LUMO levels of designed dyes P1–P6 are in the range of −2.98 to −2.79 eV. Those values lie above the conduction band (CB) of the TiO_2_ (−4.0 eV), indicating that excited state designed dyes P1–P6 could quickly and efficiently electron injections into the TiO_2_ conduction band. The HOMO levels of designed dyes P1–P6 are in the range of −4.82 to −4.93 eV. Those values are below the redox potential of $I^{-}/I_{3}^{-}$ electrolyte (−4.60 eV) [63], demonstrating that oxidized dyes could quickly get electrons from the electrolyte. According to the above analyses, those dyes are better photosensitizer candidates. As shown in Figure 3a, P1 and P4 (−4.82 eV) have the higher HOMO level, followed by P3 (−4.84 eV), P6 (−4.85 eV), P2 (−4.92 eV), P5 (−4.93 eV), and A1 (−5.08 eV), respectively. It is clearly that the HOMO levels of P1–P6 are up-shifted compared to A1, indicating that introduction of thiophene derivatives to replace NDI moiety has little effect on HOMO level. The HOMO-1 of dyes P1–P6 and A1, lying at about −5.30, −5.44, −5.33, −5.29, −5.43, −5.35, and −6.03 eV (see Table S1), which is about 0.48, 0.52, 0.49, 0.47, 0.50, 0.50, and 0.95 eV below their respective HOMO. The LUMO energy levels are in following the order: P4 (−2.79 eV), P1 (−2.84 eV), P3 (−2.85 eV), P6 (−2.87 eV), P2 (−2.90 eV), P5 (−2.98 eV), and A1 (−3.43 eV), respectively. We can find that the LUMO levels of P1–P6 are up-shifted significantly compared to A1, indicating that introduction of thiophene derivatives to replace NDI moiety has a significant effect on LUMO level. The LUMO+1 of dyes P1–P6 and A1, lying at about −1.85, −1.90, −1.91, −1.76, −2.09, −1.96, and −2.55 eV (see Table S1), which is about 0.99, 1.00, 0.94, 1.03, 0.89, 0.91, and 0.88 eV above that of LUMO. From Figure 3a we can find that the energy gaps between the HOMO and the LUMO of P1–P6 are larger than that of A1 due to the higher LUMO level.

**Figure 4:**
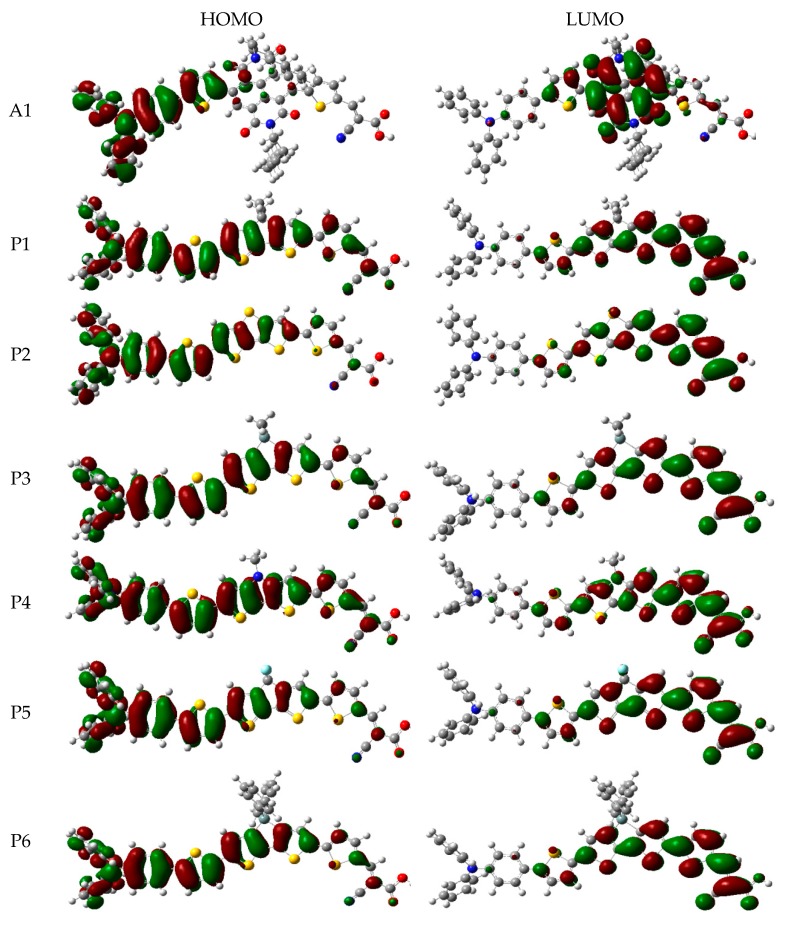
Frontier molecular orbitals of dyes P1–P6 and A1 in chloroform solvent.

**Figure 5:**
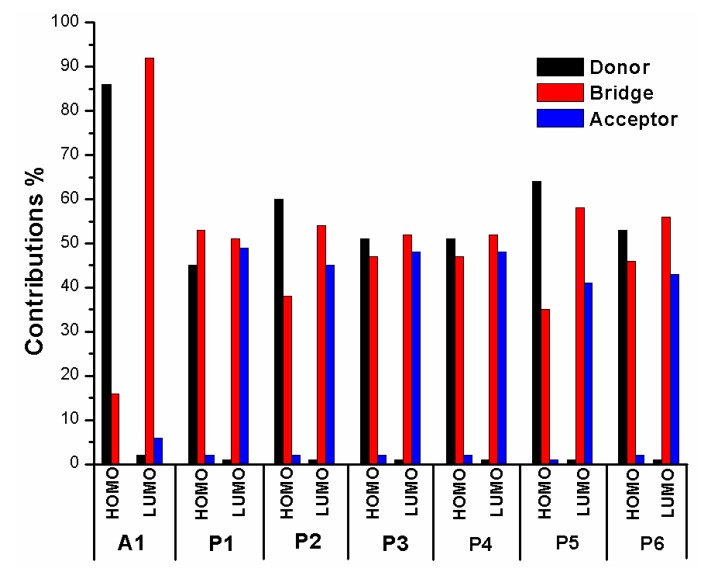
Molecular orbital compositions of the individual groups in HOMO and LUMO of P1–P6 and A1 in chloroform solvent.

In order to clearly see the electron distribution and further understand the relationship between the electronic structure and electron transition. The frontier molecular orbitals diagram (HOMO and LUMO) of P1–P6 and A1 are shown in Figure 4. Figure 4 clearly indicates that the HOMO orbital of A1 is delocalized over donor unit and the left part of π-spacer, while the LUMO orbital of A1 is mainly localized on π-spacer. For dyes P1–P6, the HOMO orbital are concentrated over donor unit and π-spacer, while the LUMO orbital are focused on π-spacer and acceptor unit. To gain more information into the frontier molecular orbitals, the molecular orbital compositions of donor, π-spacer and acceptor for HOMO and LUMO are shown in Figure 5 and the values are listed in Table S2. For dye A1, donor moiety contributes significantly (86%) of HOMO orbital, while π-spacer contributes significantly (92%) of LUMO orbital. For dyes P1–P6, the orbital densities of HOMO are mainly localized on the donor unit and π-spacer, and the values are 45%(D)/53%(π-spacer), 60%(D)/38%(π-spacer), 51%(D)/47%(π-spacer), 51%(D)/47%(π-spacer), 64%(D)/35%(π-spacer), and 53%(D)/46%(π-spacer), respectively. The orbital densities of LUMO are mainly centered at π-spacer and acceptor unit, and the values are 51%(π-spacer)/49%(A), 54%(π-spacer)/45%(A), 52%(π-spacer)/48%(A), 52%(π-spacer)/48%(A), 58%(π-spacer)/41%(A), and 56%(π-spacer)/43%(A), respectively. This better charge separation of HOMO and LUMO for dyes P1–P6 is benefit to electron injection from the excited dye to the conduction band of the semiconductor. The difference between the molecular orbital compositions (MOC) of acceptor unit was used to study the charge-transfer property. P1 exhibits highest molecular orbital compositions (MOC) difference (47%), followed by P3 = P4 (46%), P2 (43%), P6 (41%), P5 (40%), and A1 (6%), respectively. It is clear that introduction of thiophene derivatives to replace NDI moiety has a significant effect on the difference between the molecular orbital compositions (MOC) of acceptor unit. Therefore, dyes P1–P6 will have more effective intramolecular charge transfer (ICT) upon photoexcitation.

After binding on the TiO_2_ surface, in order to further investigate the electronic coupling between LUMO and the conduction band (CB) of the TiO_2_, the energy level (HOMO−2, HOMO−1, HOMO, LUMO, LUMO+1, and LUMO+2) of dye was calculated, which were introduced here because some states come from those orbitals and the results are listed in Table S1, the corresponding energy level diagram of the dyes after binding on the TiO_2_ surface is shown in Figure 3b. The HOMO levels (see Figure 3b) of dyes P1/TiO_2_–P6/TiO_2_ and A1/TiO_2_ are −4.86, −4.96, −4.88, −4.87, −4.96, −4.90, and −5.07 eV, respectively. It is clear that HOMO levels of dyes P1/TiO_2_–P6/TiO_2_ have a subtle increase compared to A1/TiO_2_. The HOMO–1 of dyes P1/TiO_2_–P6/TiO_2_ and A1/TiO_2_, lying at about, −5.37, −5.50, −5.40, −5.36, −5.50, −5.41, and −6.07 eV (see Table S1), which is about 0.51, 0.54, 0.52, 0.49, 0.54, 0.51, and 1.00 eV below their respective HOMO. Compared to the isolated dyes, the HOMO and HOMO-1 levels of dyes/TiO_2_ are almost unchanged. The LUMO levels (see Figure 3b) of dyes P1/TiO_2_–P6/TiO_2_ and A1/TiO_2_ are −3.35, −3.37, −3.35, −3.34, −3.37, −3.36, and −3.48 eV, respectively, indicating that the LUMO levels have no obvious change. The LUMO+1 levels of dyes P1/TiO_2_–P6/TiO_2_ and A1/TiO_2_, lying at about −3.24, −3.26, −3.24, −3.24, −3.26, −3.26, and −3.40 eV (see Table S1), there is no obvious change compared to their LUMO levels. Compared to the isolated dyes, the LUMO level of A1/TiO_2_ has no obvious change. For P1/TiO_2_–P6/TiO_2_, the LUMO levels are down-shifted significantly compared to isolated dyes P1–P6. The LUMO+1 levels of dyes P1/TiO_2_–P6/TiO_2_ and A1/TiO_2_ are also down-shifted significantly compared to isolated dyes. The energy gaps of dyes P1/TiO_2_–P6/TiO_2_ and A1/TiO_2_ are in the range of 1.51 to 1.59 eV, there is no obvious change (Figure 3b). After binding on the TiO_2_ surface, HOMO levels of P1/TiO_2_–P6/TiO_2_ have no obvious change compared to the isolated dyes, while the LUMO levels of P1/TiO_2_–P6/TiO_2_ are down-shifted significantly compared to isolated dyes, which leads to energy gaps decrease by 0.47, 0.43, 0.46, 0.50, 0.36, and 0.44 eV for P1/TiO_2_–P6/TiO_2_ compared to their isolated dyes (see Figure 3b), and the energy gap of A1/TiO_2_ has minor change compared to its isolated dyes. Once the dyes binding on the TiO_2_ surface, the energy levels of dyes will change due to bonding mechanism between the semiconductor conduction band and dye, therefore, some photoelectric properties of dyes/TiO_2_ will change compared to dye.

**Figure 6:**
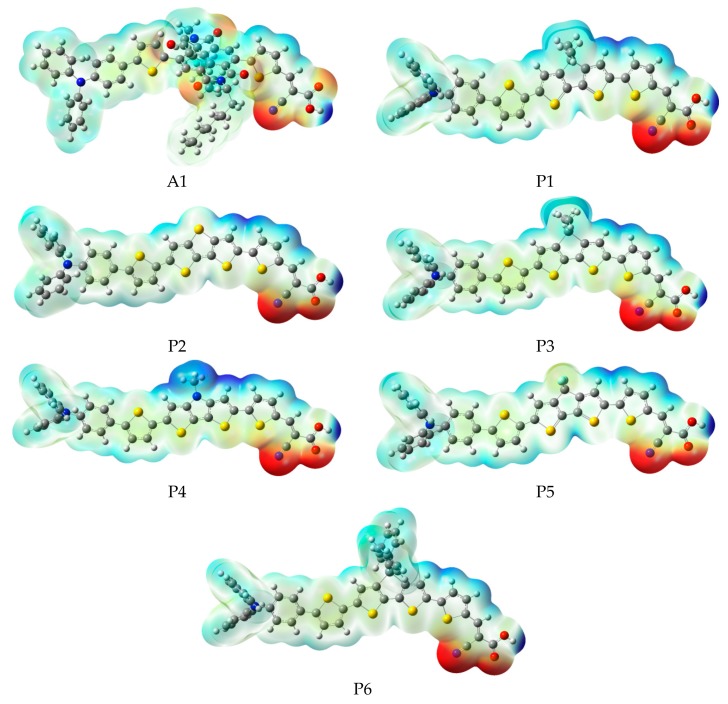
Molecular electrostatic potential plots of dyes P1–P6 and A1 in chloroform solvent.

In order to predict and explain chemical reactions (such as hydrogen bonding interactions), molecular electrostatic potential (MEP) were calculated at B3LYP/6-31G(d), which has a close relationship with the electron cloud, and the result are shown in Figure 6. Molecular electrostatic potential (MEP) is used to describe the nucleophilic and electrophilic reaction sites. The different colors at the surface represent different electrostatic potential values. The red (negative) color area of the molecular electrostatic potential (MEP) depicts electrophilic reactivity, which refer to electron-rich areas. While blue (positive) color area depicts nucleophilic reactivity, which refer to electron-poor areas. The values of electrostatic potential are in increasing order: red < orange < yellow < green < blue. The color code of molecular electrostatic potential (MEP) maps ranges from −0.06 a.u. (deepest red) to 0.06 a.u. (deepest blue). Figure 6 indicates that the most positive potential is observed on carboxyl hydrogen atom, which is preferred area for nucleophilic attack. The greatest negative potential is found on nitrogen atom of -CN group, which is the preferred site of electrophilic attack.

**Figure 7:**
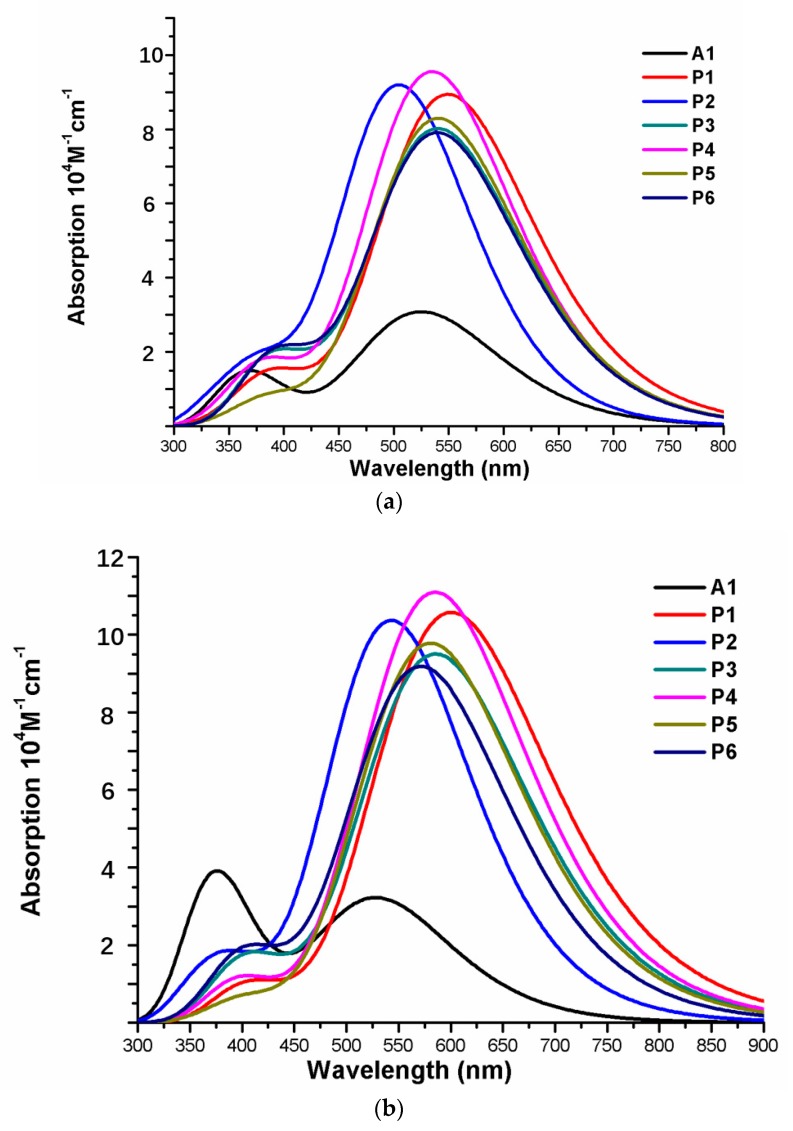
Absorption spectra of (**a**) isolated dyes P1–P6 and A1, (**b**) dyes P1–P6 and A1 after bound to TiO_2_ surface in chloroform solvent.

**Figure 8:**
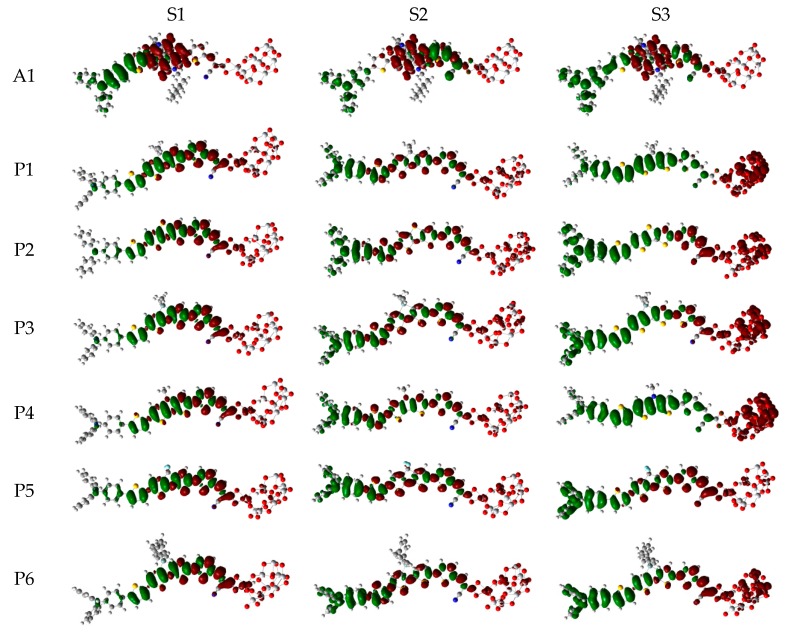
Charge difference density (CDD) of the selected excited state for dyes P1–P6 and A1 after bound to TiO_2_ surface in chloroform solvent.

Over the years, time-dependent DFT (TD-DFT) has been widely used in simulation of absorption and emission spectra [64]. Therefore, absorption spectra of dyes P1–P6 and A1 were calculated by using TD-DFT//Cam-B3LYP/6-31G(d) method based on the geometry optimization of the ground state. The functional selection is discussed in Section 2.2. The calculated oscillator strengths (f), excitation energies ($E_{g}$), maximum absorption wavelengths ($\lambda_{\max}$), and main electron transition are listed in Table 2, and the UV–vis absorption spectra with DFT results are shown in Figure 7. Table 2 illustrates that the first excited states of dyes P1–P6 and A1 show the same electronic transition from HOMO to LUMO. The maximum absorption peaks ($\lambda_{\max}$) of the dyes P1–P6 and A1 are found at 505–549 nm, which are all in visible region (see Figure 7a). It is the important region for photo-to-current conversion. The maximum absorption peaks ($\lambda_{\max}$) of dyes are in following order; P1 (549 nm) > P3 (541 nm) = P5 (541 nm) > P6 (540 nm) > P4 (535 nm) > A1 (525 nm) > P2 (505 nm). Compared to A1, it is clear that only P2 shows blue shift by 20 nm. P1, P3, P4, P5, and P6 show red shift by 24, 16, 16, 15, and 10 nm, respectively. The red-shift of the absorption peak is beneficial to improve the open-circuit photovoltage ($V_{OC}$) and short-circuit photocurrent ($J_{SC}$), which leads to a higher photoelectric conversion efficiency (PCE) of the DSSC. Absorption spectra (see Figure 7a) shows that P1–P6 exhibit a broad absorption band compared to A1 in the visible region, which is beneficial for improving the efficiency. After binding on the TiO_2_ surface, absorption properties (such as maximum absorption peak and oscillator strength) of the dyes/TiO_2_ were obviously changed due to the interaction between the dyes and the TiO_2_. The electronic and optical parameters of dyes/TiO_2_ are listed in Table 2, and the absorption spectra are shown in Figure 7b. The maximum absorption peaks ($\lambda_{\max}$) of dyes/TiO_2_ are in following order; P1/TiO_2_ (601 nm) > P3/TiO_2_ (586 nm) > P4/TiO_2_ (585 nm) > P5/TiO_2_ (581 nm) > P6/TiO_2_ (572 nm) > P2/TiO_2_ (543 nm) > A1/TiO_2_ (529 nm). It is clear that dyes P1–P6 show a significant red-shift compared to A1, and the values are 72, 57, 56, 52, 43, and 14 nm, respectively. The first excited states of all the dye/TiO_2_ show a major contribution from HOMO to LUMO. Compared to the isolated dyes, the maximum absorption peak ($\lambda_{\max}$) is 52, 38, 45, 50, 40, 32, and 4 nm red-shift for dyes P1/TiO_2_–P6/TiO_2_ and A1/TiO_2_, respectively. To analyze the charge transfer properties of the excited state for dyes/TiO_2_, the charge difference density (CDD) are shown in Figure 8, where the green and red color stand for an increase and a decrease of electronic density, respectively. As depicted in Figure 8, for A1/TiO_2_, the density increment region of the excited state S1, S2, and S3 is mostly located on NDI moiety, while the density depletion zone is mainly distributed on donor group. For P1/TiO_2_-P6/TiO_2_, the electronic density of the excited state S1 shows a decrease at π-spacer and a increase at the π-spacer and acceptor moiety, the electronic density of the excited state S2 shows a decrease at donor and π-spacer and a increase at the π-spacer, acceptor and TiO_2_. For the excited state S3, evidently, the electronic density of P1/TiO_2_ and P4/TiO_2_ are all located on TiO_2_, while the density increment zone for P2/TiO_2_, P3/TiO_2_, and P6/TiO_2_ is mostly located on TiO_2_ and rest electronic density is located on acceptor moiety. The electronic density of P5/TiO_2_ is all located on the π-spacer and acceptor moiety. In summary, the electrons in the excited state S6 for P1/TiO_2_–P4/TiO_2_ and P6/TiO_2_ are in direction form dye to TiO_2_.

The emission properties (fluorescence energies, the emission peaks and oscillator strengths and) of the dyes P1–P6 and A1 were calculated at the TD-DFT//CAM-B3LYP/6-31G(d) level in chloroform solution, and the results are listed in Table 3. As shown in Table 3, the maximum emission wavelengths ($\lambda_{em}$) of dyes exhibit higher wavelengths (~100 nm) than their maximum absorption wavelengths ($\lambda_{\max}$). P5 has largest $\lambda_{em}$ at 685 nm which is 144 nm higher than its $\lambda_{\max}$, while P2 has lowest $\lambda_{em}$ with 610 nm, which is 105 nm higher than its $\lambda_{\max}$. The maximum emission wavelengths ($\lambda_{em}$) of dyes are in following order; P5 > A1 > P6 > P3 > P1 > P4 > P2. All dyes exhibit same electronic transition from HOMO to LUMO. Compared to A1, the maximum emission wavelength of P6 shows red shift by 9 nm while P1–P5 show blue shift by 6, 66, 11, 56, and 8 nm, respectively. P6 can be used as efficient blue-light-emitting materials due to larger oscillator strength and the emission peak.

To better predict the short-circuit photocurrent ($J_{SC}$) of dyes, light harvesting efficiency (LHE) and the driving force of electron injection ($\Delta G^{inject}$) are calculated, and the results are listed in Table 4. According to Equation (3), it is clear that light harvesting efficiency (LHE) is related to the oscillator strength (f). In order to obtain larger light harvesting efficiency (LHE), the oscillator strength (f) should be as higher as possible. The oscillator strengths (f) of dyes P1–P6 are 2.2053, 2.2594, 1.9759, 2.3560, 2.0460, and 1.9468 (see Table 2), respectively, which are much higher than that of A1 (0.7597). Therefore, light harvesting efficiency (LHE) of dyes P1–P6 are larger than that of A1, and the values are in following order; P4 (0.996) > P1 (0.994) = P2 (0.994) > P5 (0.991) > P3 (0.989) = P6 (0.989) > A1 (0.826). The results show that introduction of thiophene derivatives to replace NDI moiety promote light harvesting efficiency (LHE) increase. Hence dyes P1–P6 can absorb more photons, which is beneficial for improving short-circuit photocurrent ($J_{SC}$). Another factor that influences short-circuit photocurrent ($J_{SC}$) is driving force of electron injection ($\Delta G^{inject}$). As shown in Table 4, the values of $\Delta G^{inject}$ were negative, indicating that excited state dye lies above the conduction band (CB) of the TiO_2_, and thus promote electron injection from the excited sensitizer to the TiO_2_ conduction band. The calculated driving force of electron injection ($\Delta G^{inject}$) are in following order; A1 > P5 > P1 = P6 > P3 > P4 > P2. Dyes P1–P6 exhibit more negative driving force of electron injection ($\Delta G^{inject}$) than that of A1, suggesting that dyes P1–P6 will exhibit faster electron injection. At the same time, the $\phi_{inj}$ of dyes P1–P6 are larger than that of MA-2 due to their more negative driving force of electron injection ($\Delta G^{inject}$). Therefore, dyes P1–P6 will exhibit larger short-circuit photocurrent ($J_{SC}$) than that of A1 according to Equation (2). The reorganization energy ($\Delta G_{reg}$) is another important factor that affects the short-circuit photocurrent ($J_{SC}$) of the DSSCs. Lower reorganization energy ($\Delta G_{reg}$) will lead to faster electron transfer. The reorganization energy ($\Delta G_{reg}$) can be expressed as [65];

(8) $$\Delta G_{reg}=E(I_{3}^{-}/I^{-})-E_{dye}$$

where $E(I_{3}^{-}/I^{-})$ is the redox potential (−4.60 eV). As shown in Table 4, the reorganization energy ($\Delta G_{reg}$) of dyes P1–P6 are in the range of 0.22 to 0.33 eV, which are lower than A1 (0.48 eV). Hence dyes P1–P6 will have higher power conversion efficiency due to the lower reorganization energy ($\Delta G_{reg}$) [66]. Above all, introduction of thiophene derivatives to replace NDI moiety can improve short-circuit photocurrent ($J_{SC}$).

In order to analyze the relationship between the LUMO and open-circuit photovoltage ($V_{OC}$), $V_{OC}$ can be approximately expressed by the formula [67]

(9) $$eV_{OC}=E_{LUMO}-E_{CB}$$

In order to obtain larger $eV_{OC}$, $E_{LUMO}$ should be as higher as possible [68,69]. As shown in Table 4, the $eV_{OC}$ values of dyes are in following order; P4 (1.21eV) > P1 (1.16 eV) > P3 (1.15 eV) > P6 (1.13 eV) > P2 (1.10 eV) > P5 (1.02 eV) > A1 (0.57 eV). It is clear that dyes P1–P6 should have a larger $V_{OC}$ compared to A1. Introduction of thiophene derivatives to replace NDI moiety not only increases short-circuit photocurrent ($J_{SC}$), but also enhances open-circuit photovoltage ($V_{OC}$).

After binding on the TiO_2_ surface, the oscillator strengths (f) of dyes P1/TiO_2_-P6/TiO_2_ are in the range of 2.7395 to 2.2638, which are greatly higher than that of A1/TiO_2_ (0.7923) (see Table 2). The light harvesting efficiency (LHE) can be calculated according to Equation (3), and the light harvesting efficiency (LHE) values of dyes/TiO_2_ are in following order; P1/TiO_2_ = P4/TiO_2_ > P2/TiO_2_ > P5/TiO_2_ > P3/TiO_2_ = P6/TiO_2_ > A1/TiO_2_. Hence dyes P1/TiO_2_–P6/TiO_2_ can absorb more photons compared to A1/TiO_2_. The oscillator strengths (f) of dyes/TiO_2_ are larger than that of the isolated dyes (see Table 2), which leads to higher light harvesting efficiency (LHE) compared to the isolated dyes (see Table 3). The driving force of electron injection ($\Delta G^{inject}$) of dyes P1/TiO_2_-P6/TiO_2_ are in the range of −1.17 to −1.32 eV, there is no obvious change compared to A1/TiO_2_ (−1.23 eV). Compared to the isolated dyes, the driving force of electron injection ($\Delta G^{inject}$) of dyes/TiO_2_ decreases slightly. The reorganization energy ($\Delta G_{reg}$) of dyes/TiO_2_ are in following order; A1/TiO_2_ > P2/TiO_2_ = P5/TiO_2_ > P6/TiO_2_ >P3/TiO_2_ >P4/TiO_2_ > P1/TiO_2_. Therefore, dyes P1/TiO_2_-P6/TiO_2_ will have higher power conversion efficiency due to the lower reorganization energy ($\Delta G_{reg}$). Compared to the isolated dyes, the reorganization energy ($\Delta G_{reg}$) of dyes/TiO_2_ has no obvious change. The $eV_{OC}$ values of dyes are in the range of 0.63 to 0.66 eV, which are slightly higher than that of A1/TiO_2_ (0.52 eV). The $eV_{OC}$ value of A1 has no obvious change before and after binding on the TiO_2_ surface. For dyes P1–P6, the $eV_{OC}$ values of dyes/TiO_2_ are slightly lower than that of isolated dyes.

### 3.2. Screening of the Donors

On the basis of A1, we designed four dyes (see Figure 1) by introducing different electron-donating groups on the triphenylamine donor of A1 (such as $-CH_{3},-NH_{2},-OCH_{3}$, and $-C_{4}H_{9}$). As shown in Table 1, it is clear that the dihedral angles ($\Phi_{1}$, $\Phi_{2}$, $\Phi_{3}$, and $\Phi_{4}$) and bond lengths ($d_{1}$, $d_{2}$, $d_{3}$, and $d_{4}$) of dyes D1–D4 have no obvious change compared to A1, which means that introduction different electron-donating groups on the triphenylamine donor of A1 has no obvious effect on dihedral angles and bond lengths.

**Figure 9:**
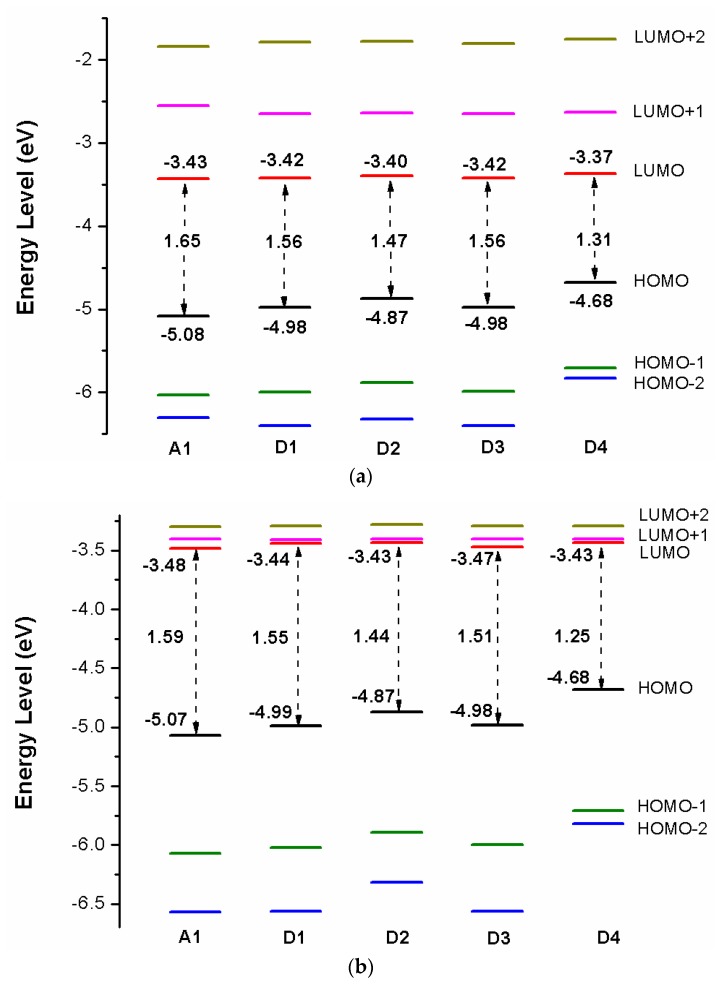
Energy level diagram of (**a**) isolated dyes D1–D4 and A1, (**b**) dyes D1–D4 and A1 after bound to TiO_2_ surface in chloroform solvent.

Figure 9a shows the energy level diagrams of dyes D1–D4 and A1 in chloroform solvent. The LUMO levels of designed dyes D1–D4 are −3.42, −3.40, −3.42, and −3.37 eV, respectively, which are higher than conduction band (CB) of the TiO_2_ (−4.0 eV). Therefore, electron injection can easily take place from the excited dyes to the TiO_2_ conduction band. Compared to A1 (−3.43 eV), the LUMO levels of dyes D1–D4 are in the range of −3.42 to −3.37 eV, which have no obvious change. Introduction different electron-donating groups on the triphenylamine donor of A1 have no obvious effect on LUMO level. The LUMO+1 of dyes D1–D4, lying at about −2.65, −2.64, −2.65, and −2.63 eV, (see Table S3), which is about 0.77, 0.76, 0.77, and 0.74 eV above that of LUMO. The HOMO levels of designed dyes D1–D4 are −4.98, −4.87, −4.98, and −4.68 eV, respectively, which are lower than redox potential of $I^{-}/I_{3}^{-}$ electrolyte (−4.60 eV). Hence oxidized dyes could quickly get electrons from the electrolyte. Compared to A1 (−5.08 eV), the HOMO levels of dyes D1–D4 are in the range of −4.98 to −4.68 eV, which have a suitable up-shift. In particular, introduction $-OCH_{3}$ (D2) and $-NH_{2}$ (D4) groups on the triphenylamine donor of A1 have a significant on HOMO level. The HOMO-1 of dyes D1–D4, lying at about −6.00, −5.88, −5.99, and −5.71 eV, (see Table S1), which is about 1.02, 1.01, 1.01, and 1.03 eV below their respective HOMO. The energy levels (HOMO and LUMO) of dyes D1–D4 match the redox potential of the $I^{-}/I_{3}^{-}$ couple and the conduction band edge level of TiO_2_. Hence dyes D1–D4 are suitable candidates for DSSCs. The energy gap between highest occupied molecular orbitals (HOMOs) and lowest unoccupied molecular orbitals (LUMOs) is an important factor affecting power conversion efficiency when the free energy of the transition, which directs the rate of charge transfer (CT). A smaller energy gap can generate more electrons in the visible and exhibits high extinction, leading to higher open-circuit photovoltage ($V_{OC}$) for better PCE of DSSCs. From Figure 9a the energy gaps between the HOMO and the LUMO of D1–D4 are lower than that of A1 due to the decrease of HOMO energy levels.

**Figure 10:**
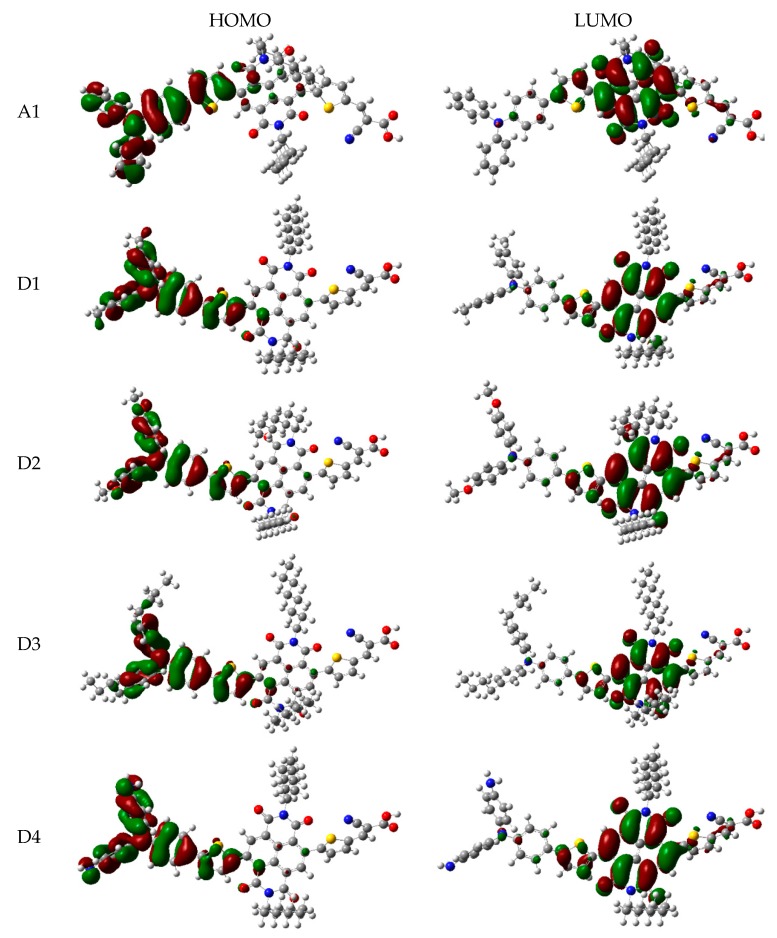
Frontier molecular orbitals of dyes D1–D4 and A1 in chloroform solvent.

**Figure 11:**
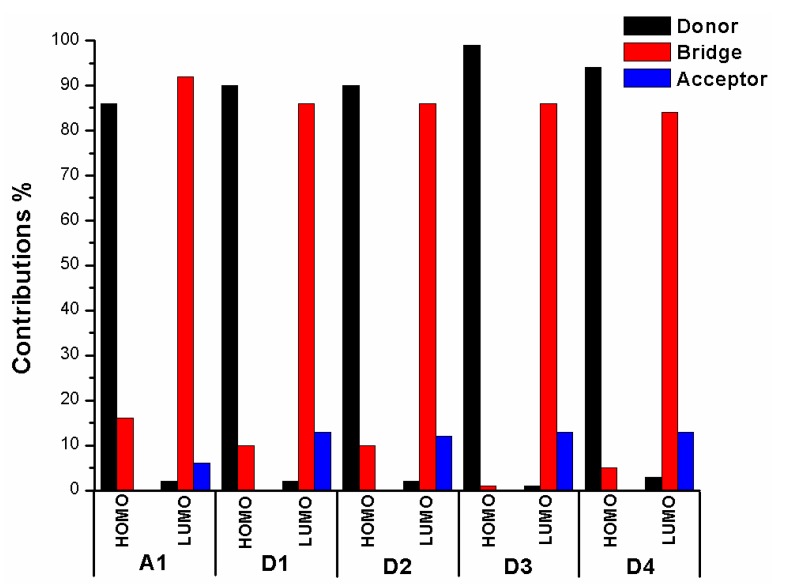
Molecular orbital compositions of the individual groups in HOMO and LUMO of D1–D4 and A1 in chloroform solvent.

The frontier molecular orbitals diagram (HOMO and LUMO) of D1–D4 and A1 are shown in Figure 10. It is obvious that the HOMO orbital of D1–D4 is delocalized over donor unit and the left part of π-spacer, while the LUMO orbital of D1–D4 is mainly localized on π-spacer. D1–D4 have similar electron densities, indicating that introduction of different electron-donating groups has no significant effect on frontier molecular orbital. To gain more information into the frontier molecular orbitals, the molecular orbital compositions of donor, π-spacer, and acceptor for HOMO and LUMO are shown in Figure 11 and the values are listed in Table S2. For HOMO, the orbital densities of D1–D4 are mainly localized on the D part, while a very small amount of orbital densities are focused in π-spacer part, and the values are 90%(D)/10%(π-spacer), 90%(D)/10%(π-spacer), 99%(D)/1%(π-spacer), and 94%(D)/5%(π-spacer), respectively. Compared to A1 86%(D)/16%(π-spacer), it is clear that orbital densities of D part increase, while orbital densities of π-spacer part decrease after introduction different electron-donating groups on the triphenylamine donor. For LUMO, the orbital densities of D1–D4 are mainly localized on the π-spacer part, while a small amount of orbital densities are focused in A part, and the values are 86%(D)/13%(π-spacer), 86%(D)/12%(π-spacer), 86%(D)/13%(π-spacer), and 84%(D)/13%(π-spacer), respectively. Compared to A1 92%(D)/6%(π-spacer), it is clear that orbital densities of π-spacer part decrease, while orbital densities of A part increase after introduction different electron-donating groups on the triphenylamine donor. D1, D3, and D4 have highest MOC difference (13%), followed by D2 (12%), which are higher than that of A1 (6%). Hence, D1–D4 exhibit better charge-transfer property compared to A1.

**Figure 12:**
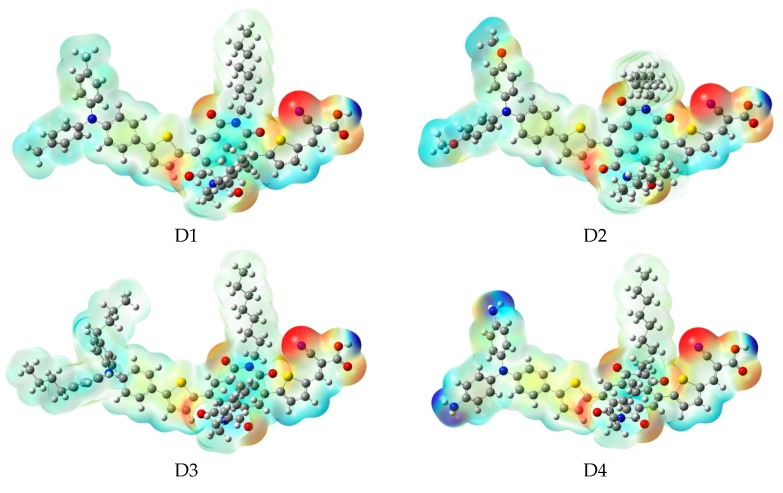
Molecular electrostatic potential plots of dyes D1–D4 in chloroform solvent.

After binding on the TiO_2_ surface, the energy level of dye was calculated, and the results are listed in Table S1, the corresponding energy level diagram is shown in Figure 9b. The HOMO levels (see Figure 9b) of dyes D1/TiO_2_–D4/TiO_2_ are −4.99, −4.87, −4.98, and −4.68 eV, respectively. Compared to A1/TiO_2_ (−5.07 eV), D1 and D3 have no obvious change, D2 and D4 have a subtle increase. The HOMO-1 of dyes D1/TiO_2_-D4/TiO_2_, lying at about, −6.02, −5.89, −6.00, and −5.71 eV (see Table S1), which is about 1.03, 1.02, 1.02, and 1.03 eV below their respective HOMO. The HOMO and HOMO-1 levels of dyes D1–D4 are almost unchanged before and after binding on the TiO_2_ surface. The LUMO levels (see Figure 9b) of dyes D1/TiO_2_-D4/TiO_2_ are −3.44, −3.43, −3.47, and −3.43 eV, respectively, indicating that the LUMO levels have no obvious change. The LUMO+1 levels of dyes D1/TiO_2_-D4/TiO_2_, lying at about −3.41, −3.40, −3.40, and −3.40 eV (see Table S1), there is no obvious change compared to their LUMO levels. Compared to the isolated dyes, the LUMO level of dyes/TiO_2_ has no obvious change. The energy gaps of dyes D1/TiO_2_–D4/TiO_2_ are 1.55, 1.44, 1.51, and 1.25 eV, respectively. The energy gaps of dyes D2/TiO_2_ and D4/TiO_2_ are lower than that of A1/TiO_2_ due to the higher HOMO energy level. Compared to their isolated dyes, the energy gap of dyes/TiO_2_ has no obvious change. Molecular electrostatic potential (MEP) are shown in Figure 12. As shown in Figure 12, the most positive potential is found on carboxyl hydrogen atom, which is preferred area for nucleophilic attack. While the greatest negative potential is observed on nitrogen atom of -CN group, which is the preferred site of electrophilic attack.

**Figure 13:**
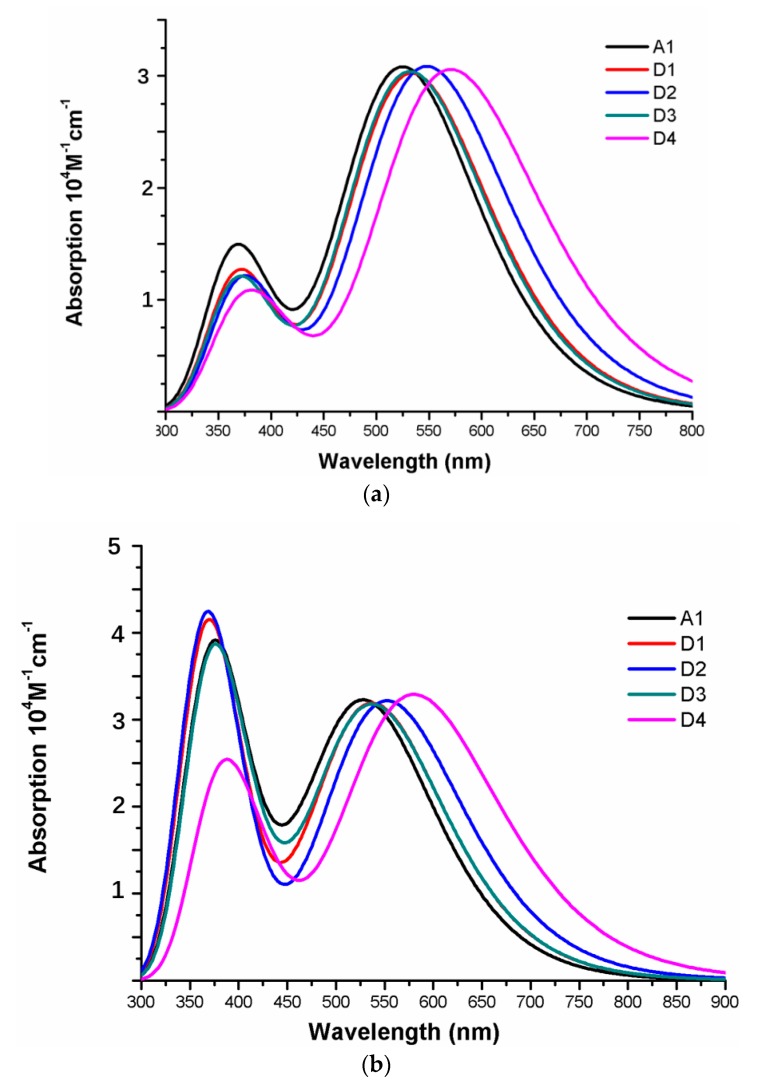
Absorption spectra of (**a**) isolated dyes D1–D4 and A1, (**b**) dyes D1–D4 and A1 after bound to TiO_2_ surface in chloroform solvent.

**Figure 14:**
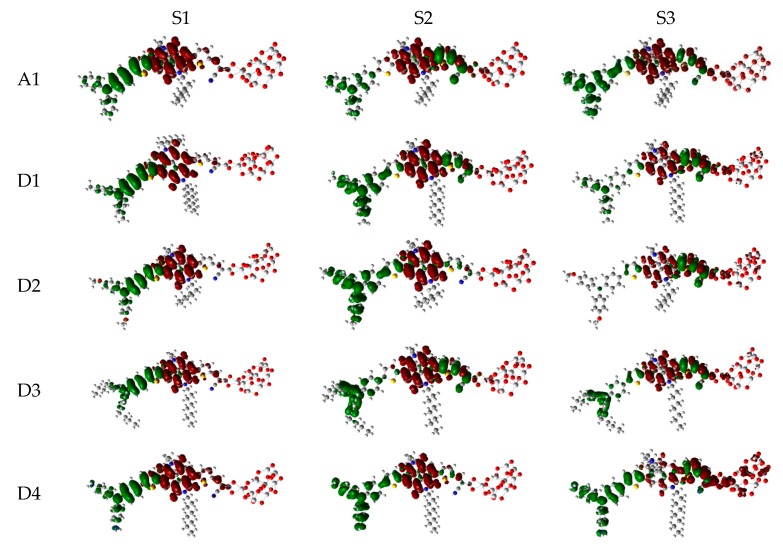
Charge difference density (CDD) of the selected excited state for dyes D1–D4 and A1 after bound to TiO_2_ surface in chloroform solvent.

The photophysical parameters of dyes D1–D4 are listed in Table 2, and the UV–vis absorption spectra are shown in Figure 13a. The first excited states of dyes D1–D4 show a transition from the HOMO to the LUMO. The maximum absorption peaks ($\lambda_{\max}$) of dyes are in following order; D4 (571 nm) > D2 (548 nm) > D1 (534 nm) > D3 (532 nm), which exhibit 46, 23, 9, and 7 nm red shift compared to that of A1, respectively. After binding on the TiO_2_ surface, the excited state properties and absorption spectra are shown in Table 2 and Figure 13b. The maximum absorption peaks ($\lambda_{\max}$) of dyes/TiO_2_ are in following order; D4/TiO_2_ (581 nm) > D2/TiO_2_ (553 nm) > D1/TiO_2_ (538 nm) = D3/TiO_2_ (538 nm), which exhibit 52, 24, 9, and 9 nm red shift compared to that of A1/TiO_2_, respectively. The first excited states of all the dye/TiO_2_ show a major contribution from HOMO to LUMO. Compared to the isolated dyes, the maximum absorption peak ($\lambda_{\max}$) is 4, 5, 6, and 10 nm red-shift for dyes D1/TiO_2_–D4/TiO_2_, respectively. As shown in Figure 14, the charge difference density (CDD) of D1/TiO_2_–D4/TiO_2_ has a similar electronic distribution compared to A1/TiO_2_. It is worth mentioning that the density increment region of the excited state S3 for D4/TiO_2_ is mainly distributed on acceptor and TiO_2_, indicating that a better intramolecular charge transfer when the transition occurs compared to A1/TiO_2_.

As shown in Table 3, the maximum emission wavelengths ($\lambda_{em}$) of dyes exhibit higher wavelengths (~150 nm) than their maximum absorption wavelengths ($\lambda_{\max}$). D4 has largest $\lambda_{em}$ at 723 nm which is 152 nm higher than its $\lambda_{\max}$. D2 exhibit $\lambda_{em}$ with 697 nm, which is 149 nm higher than its $\lambda_{\max}$. While D1 and D3 have lowest $\lambda_{em}$ with 685 nm, which is 151 and 153 nm higher than its $\lambda_{\max}$. All dyes exhibit same electronic transition from HOMO to LUMO. Compared to A1, the maximum emission wavelength of D1–D4 show red shift by 9, 21, 9, and 47 nm, respectively.

The oscillator strength (f) is an important factor affecting the light harvesting efficiency (LHE). As shown in Table 4, the oscillator strengths (f) of D1–D4 are in the range of 0.7459-0.7609, which have no obvious change compared to A1 (0.7597). Hence light harvesting efficiency (LHE) of D1–D4 has no obvious change, and the values are 0.820, 0.827, 0.822, and 0.824, respectively. The calculated driving force of electron injection ($\Delta G^{inject}$) are in following order; D1 > D3 > D2 > D4, which are more negative than that of A1, suggesting that dyes D1–D4 will exhibit faster electron injection and larger short-circuit photocurrent ($J_{SC}$). The reorganization energy ($\Delta G_{reg}$) of dyes D1–D4 are 0.38, 0.27, 0.38, and 0.08 eV, respectively, which are lower than A1 (0.48 eV). Hence dyes P1–P6 will have higher power conversion efficiency due to the lower reorganization energy ($\Delta G_{reg}$). The $eV_{OC}$ values of D1–D4 are in the range of 0.58 to 0.63 eV, which have no obvious change compared to A1 (0.57 eV).

After binding on the TiO_2_ surface, the oscillator strengths (f) of D1/TiO_2_-D4/TiO_2_ are in the range of 0.7819-0.8108, which have no obvious change compared to A1 (0.7923). The light harvesting efficiency (LHE) of D1/TiO_2_-D4/TiO_2_ are 0.836, 0.839, 0.835, and 0.845, respectively. There is no obvious change compared to A1/TiO_2_ (0.839). The driving force of electron injection ($\Delta G^{inject}$) of D1/TiO_2_–D4/TiO_2_ are more negative than that of A1/TiO_2_, D2/TiO_2_ exhibit most negative $\Delta G^{inject}$ (−2.51 eV). The $eV_{OC}$ values of D1/TiO_2_–D4/TiO_2_ have no obvious change compared to A1/TiO_2_. D4/TiO_2_ has lowest $\Delta G_{reg}$, followed by D2/TiO_2_, D3/TiO_2_, and D1/TiO_2_. Compared to the isolated dyes, the driving force of electron injection ($\Delta G^{inject}$) of D1/TiO_2_ and D2/TiO_2_ increases slightly, while D3/TiO_2_ and D4/TiO_2_ have no obvious change. The light harvesting efficiency (LHE), reorganization energy ($\Delta G_{reg}$) and $eV_{OC}$ of D1–D4 have no obvious change before and after binding on the TiO_2_ surface.

## 4. Conclusions

In this work, we have systematically investigated optical and electrical properties of D-π-A type dyes designed by introducing different functional groups on the donor and π-spacer. The results indicate that π-spacer in P1–P6 exhibit rigid structures, which will enhance electrons more easily transferred from donor to acceptor. P1 and P3–P6 show red shift compared to A1. In particular, light harvesting efficiency (LHE), driving force of electron injection ($\Delta G^{inject}$), reorganization energy ($\Delta G_{reg}$), and $eV_{OC}$ of P1–P6 which related to the short-circuit current density ($J_{SC}$) and open-circuit photovoltage ($V_{OC}$), are superior to those of A1. Therefore, P1–P6 are regarded as outstanding candidates for use in DSSCs. Furthermore, the donor group in D2 and D4 exhibit lower energy gap and great red shift compared to A1. D4 shows the more driving force of electron injection ($\Delta G^{inject}$) and lower reorganization energy ($\Delta G_{reg}$) among the D1–D4, which are superior to those of A1. This is beneficial to improving the short-circuit current density ($J_{SC}$). Above all, P1–P6 and D4 are promising organic dyes and display better photoelectric performance in rational molecular engineering of sensitizers combining high photovoltaic efficiency.

## Figures and Tables

**Table 1:** Selected bond lengths (Å) and dihedral angles (°) of triphenylamine derivative dyes in chloroform solvent.

	$\Phi_{1}$	$\Phi_{2}$	$\Phi_{3}$	$\Phi_{4}$	$d_{1}$	$d_{2}$	$d_{3}$	$d_{4}$
A1	22.46	−37.79	−118.77	−179.70	1.460	1.465	1.481	1.425
P1	−21.17	9.48	0.20	−179.94	1.462	1.441	1.433	1.415
P2	21.30	−9.82	−1.96	−0.25	1.461	1.442	1.438	1.419
P3	−20.56	7.54	1.84	−179.72	1.462	1.442	1.435	1.416
P4	−20.53	8.76	0.35	179.86	1.462	1.441	1.433	1.415
P5	−21.06	11.17	4.81	179.93	1.462	1.441	1.436	1.418
P6	−18.34	8.75	1.33	−179.92	1.462	1.442	1.436	1.417
D1	21.74	−38.95	−117.02	−179.63	1.460	1.465	1.482	1.425
D2	23.62	−36.97	−118.20	−179.94	1.459	1.463	1.481	1.425
D3	21.71	−38.04	−115.34	−179.49	1.461	1.464	1.482	1.425
D4	22.15	−36.17	−116.88	−179.67	1.457	1.462	1.481	1.424

**Table 2:** Computed excitation energy ($E_{g}$/eV), maximum absorption wavelength ($\lambda_{\max}$/nm), oscillator strength (f), and electronic transition configuration of triphenylamine derivative dyes in chloroform solution.

Dye	State	$E_{g}$ $/\lambda_{\max}$	f	Main Configurations
A1	1	2.36/525	0.7597	H → L/0.58940
	2	3.26/380	0.1511	H-2 → L/0.43402
	3	3.43/362	0.2330	H-2 → L/0.40002
P1	1	2.26/549	2.2053	H → L/0.59065
	2	3.15/393	0.3574	H → L + 1/0.51442
	3	3.58/347	0.0434	H-1 → L/0.37490
P2	1	2.45/505	2.2594	H → L/0.51866
	2	3.21/386	0.4115	H → L + 1/0.48619
	3	3.66/338	0.1640	H → L/0.42560
P3	1	2.29/541	1.9759	H → L/0.56296
	2	3.15/394	0.4839	H → L + 1/0.49594
	3	3.56/348	0.0119	H → L/0.36012
P4	1	2.32/535	2.3560	H → L/0.59699
	2	3.20/388	0.4152	H → L + 1/0.53467
	3	3.59/345	0.0676	H-2 → L/0.57213
P5	1	2.29/541	2.0460	H → L/0.52808
	2	3.14/395	0.1942	H → L + 1/0.45139
	3	3.50/354	0.0343	H-1 → L + 1/0.37664
P6	1	2.29/540	1.9468	H → L/0.55318
	2	3.13/396	0.5035	H → L + 1/0.48466
	3	3.54/351	0.0117	H → L/0.36083
D1	1	2.32/534	0.7459	H → L/0.59261
	2	3.24/382	0.1267	H-1 → L/0.46484
	3	3.40/365	0.1976	H-2 → L/0.48305
D2	1	2.26/548	0.7609	H → L/0.59379
	2	3.18/390	0.1150	H-1 → L/0.48838
	3	3.37/368	0.2005	H-2 → L/0.52160
D3	1	2.33/532	0.7493	H → L/0.58869
	2	3.24/382	0.1095	H-1 → L/0.47235
	3	3.39/365	0.1981	H-2 → L/0.50056
D4	1	2.17/571	0.7540	H → L/0.59999
	2	3.06/405	0.1200	H-1 → L/0.51377
	3	3.36/369	0.1856	H-3 → L/0.55893
A1/TiO_2_	1	2.34/529	0.7923	H → L/0.58409
	2	3.23/384	0.5380	H-1 → L/0.36516
	3	3.38/366	0.4640	H → L/0.37280
P1/TiO_2_	1	2.06/601	2.6104	H → L/0.54964
	2	3.02/411	0.2565	H → L + 16/0.35495
	3	3.29/376	0.0087	H → L + 1/00.55205
P2/TiO_2_	1	2.28/543	2.5567	H → L/0.48438
	2	3.12/398	0.3216	H → L + 16/0.36183
	3	3.46/359	0.2092	H → L/0.43632
P3/TiO_2_	1	2.12/586	2.3440	H → L/0.51644
	2	3.02/410	0.4186	H-1 → L/0.32626
	3	3.33/372	0.0355	H → L/0.41457
P4/TiO_2_	1	2.12/585	2.7395	H → L/0.53415
	2	3.09/401	0.2884	H-1 → L/0.32472
	3	3.30/376	0.0030	H → L + 1/0.57187
P5/TiO_2_	1	2.13/581	2.4142	H → L/0.50933
	2	3.01/412	0.1571	H-1 → L/0.34133
	3	3.32/373	0.0276	H → L/0.41752
P6/TiO_2_	1	2.17/572	2.2638	H → L/0.49491
	2	3.04/407	0.4603	H-2 → L/0.30426
	3	3.36/369	0.0316	H → L/0.45448
D1/TiO_2_	1	2.31/538	0.7861	H → L/0.59277
	2	3.26/380	0.2399	H-1 → L/0.48023
	3	3.38/367	0.8011	H-2 → L + 4/0.35860
D2/TiO_2_	1	2.24/553	0.7936	H → L/0.59502
	2	3.19/388	0.1238	H-1 → L/0.51121
	3	3.38/367	0.9464	H-3 → L/0.41438
D3/TiO_2_	1	2.31/538	0.7819	H → L/0.58538
	2	3.21/387	0.3757	H-1 → L/0.44284
	3	3.35/370	0.6066	H-2 → L + 4/0.30048
D4/TiO_2_	1	2.13/581	0.8108	H → L/0.59550
	2	3.01/411	0.2016	H-1 → L/0.50064
	3	3.26/380	0.4754	H → L + 4/0.37904

**Table 3:** Emission properties of triphenylamine derivative dyes in chloroform solvent.

Dye	State	$E_{flu}$ $\left(\mathrm{eV})/\lambda_{em}(\mathrm{nm}\right)$	f	Main Configurations
A1	1	1.84/676	0.7606	H → L/0.64473
P1	1	1.87/662	2.3909	H → L/0.65705
P2	1	2.03/610	2.4533	H → L/0.62022
P3	1	1.86/665	2.1311	H → L/0.64954
P4	1	2.00/620	2.5404	H → L/0.64919
P5	1	1.81/685	2.1693	H → L/0.64219
P6	1	1.86/668	2.0739	H → L/0.64715
D1	1	1.81/685	0.7513	H → L/0.64168
D2	1	1.78/697	0.7130	H → L/0.63564
D3	1	1.81/685	0.7540	H → L/0.64135
D4	1	1.72/723	0.7148	H → L/0.62513

**Table 4:** Calculated electronic properties of triphenylamine derivative dyes.

Dye	LHE	$\Delta G^{inject}$	$E_{dye*}$	$E_{dye}$	$\Delta G_{reg}$	$eV_{OC}$
A1	0.826	−1.28	2.72	5.08	0.48	0.57
P1	0.994	−1.44	2.56	4.82	0.22	1.16
P2	0.994	−1.53	2.47	4.92	0.32	1.10
P3	0.989	−1.45	2.55	4.84	0.24	1.15
P4	0.996	−1.50	2.50	4.82	0.22	1.21
P5	0.991	−1.36	2.64	4.93	0.33	1.02
P6	0.989	−1.44	−2.56	4.85	0.25	1.13
D1	0.820	−1.34	2.66	4.98	0.38	0.58
D2	0.827	1.39	2.61	4.87	0.27	0.60
D3	0.822	−1.35	2.65	4.98	0.38	0.58
D4	0.824	−1.49	2.51	4.68	0.08	0.63
A1/TiO_2_	0.839	−1.23	−2.77	5.07	0.47	0.52
P1/TiO_2_	0.998	−1.20	−2.80	4.86	0.22	0.65
P2/TiO_2_	0.997	−1.32	−2.68	4.96	0.36	0.63
P3/TiO_2_	0.995	−1.24	−2.76	4.88	0.28	0.65
P4/TiO_2_	0.998	−1.25	−2.75	4.87	0.27	0.66
P5/TiO_2_	0.996	−1.17	−2.83	4.96	0.36	0.63
P6/TiO_2_	0.995	−1.27	−2.73	4.90	0.30	0.64
D1/TiO_2_	0.836	−2.39	1.61	4.99	0.39	0.56
D2/TiO_2_	0.839	−2.51	1.49	4.87	0.27	0.57
D3/TiO_2_	0.835	−1.33	2.67	4.98	0.38	0.53
D4/TiO_2_	0.845	−1.45	2.55	4.68	0.08	0.57

## Supplementary Materials

The following are available online at http://www.mdpi.com/1996-1944/12/1/193/s1, Table S1: HOMO, LUMO, and energy gap (in eV) of triphenylamine derivative dyes computed at the B3LYP/6-31G(d) level; Table S2: The FMO composition (%) of the individual groups of triphenylamine derivative dyes.
