# Supplementary material for: Design, Electron Transfer Process, and Opto-Electronic Property of Solar Cell Using Triphenylamine-Based D-π-A Architectures
Source: Materials (Basel). 2019 Jan 8;12(1):193. doi: 10.3390/ma12010193 (PMC6337444; doi:10.3390/ma12010193)
Supplement: Supplementary file 1 [file materials-12-00193-s001.pdf]

materials

Supplementary

Design, Electron Transfer Process, and Opto-Electronic Property of Solar Cell Using Triphenylamine-Based D-π-A Architectures

Yuanchao Li [1], Lu Mi [1], Haibin Wang [1], Yuanzuo Li [1,*], and Jianping Liang [2,*]

[1] College of Science, Northeast Forestry University, Harbin 150040, China; liyuanchao2018@126.com (Y.L.); milufine@sina.com (L.M.); whbhit@126.com (H.W.)

[2] Key Lab of Materials Modification, Ministry of Education, Dalian University of Technology, Dalian 116024, China

* Correspondence: yzli@nefu.edu.cn (Y.L.); liangjp0601@mail.dlut.edu.cn (J.L.); Tel.: +86-451-82192245 8211

Received: 29 November 2018; Accepted: 28 December 2018; Published: 8 January 2019

Table S1. HOMO, LUMO and energy gap (in eV) of triphenylamine derivative dyes computed at the B3LYP/6-31G(d) level.

	HOMO-2	HOMO-1	HOMO	LUMO	LUMO+1	LUMO+2	E_g
A1	-6.30	-6.03	-5.08	-3.43	-2.55	-1.84	1.65
P1	-6.08	-5.30	-4.82	-2.84	-1.85	-1.08	1.98
P2	-6.18	-5.44	-4.92	-2.90	-1.90	-1.11	2.02
P3	-6.07	-5.33	-4.84	-2.85	-1.91	-1.19	1.99
P4	-6.00	-5.29	-4.82	-2.79	-1.76	-0.96	2.03
P5	-6.19	-5.43	-4.93	-2.98	-2.09	-1.34	1.95
P6	-6.09	-5.35	-4.85	-2.87	-1.96	-1.25	1.98
D1	-6.40	-6.00	-4.98	-3.42	-2.65	-1.79	1.56
D2	-6.32	-5.88	-4.87	-3.40	-2.64	-1.78	1.47
D3	-6.40	-5.99	-4.98	-3.42	-2.65	-1.81	1.56
D4	-5.83	-5.71	-4.68	-3.37	-2.63	-1.75	1.31
A1/TiO_2	-6.57	-6.07	-5.07	-3.48	-3.40	-3.30	1.59
P1/TiO_2	-6.17	-5.37	-4.86	-3.35	-3.24	-3.17	1.51
P2/TiO_2	-6.25	-5.50	-4.96	-3.37	-3.26	-3.20	1.59
P3/TiO_2	-6.16	-5.40	-4.88	-3.35	-3.24	-3.18	1.53
P4/TiO_2	-6.08	-5.36	-4.87	-3.34	-3.24	-3.17	1.53
P5/TiO_2	-6.27	-5.50	-4.96	-3.37	-3.26	-3.22	1.59
P6/TiO_2	-6.18	-5.41	-4.90	-3.36	-3.26	-3.19	1.54
D1/TiO_2	-6.56	-6.02	-4.99	-3.44	-3.41	-3.29	1.55
D2/TiO_2	-6.32	-5.89	-4.87	-3.43	-3.40	-3.28	1.44
D3/TiO_2	-6.56	-6.00	-4.98	-3.47	-3.40	-3.29	1.51
D4/TiO_2	-5.82	-5.71	-4.68	-3.43	-3.40	-3.29	1.25

Table S2. The FMO composition (%) of the individual groups of triphenylamine derivative dyes

Dye		H	L
	D	86	2
A1	π-spacer	16	92
	A	0	6
	D	45	1
P1	π-spacer	53	51
	A	2	49
	D	60	1
P2	π-spacer	38	54
	A	2	45
	D	51	1
P3	π-spacer	47	52
	A	2	48
	D	51	1
P4	π-spacer	47	52
	A	2	48
	D	64	1
P5	π-spacer	35	58
	A	1	41
	D	53	1
P6	π-spacer	46	56
	A	2	43
	D	90	2
D1	π-spacer	10	86
	A	0	13
	D	90	2
D2	π-spacer	10	86
	A	0	12
	D	99	1
D3	π-spacer	1	86
	A	0	13
	D	94	3
D4	π-spacer	5	84
	A	0	13
